# Preservation of Autophagy May Be a Mechanism Behind Healthy Aging

**DOI:** 10.1111/acel.70246

**Published:** 2025-10-13

**Authors:** Arsun Bektas, Shepherd H. Schurman, Julián Candia, Olaya Santiago‐Fernández, Susmita Kaushik, Ana Maria Cuervo, Luigi Ferrucci

**Affiliations:** ^1^ Translational Gerontology Branch National Institute on Aging, National Institutes of Health Baltimore Maryland USA; ^2^ Clinical Research Core National Institute on Aging, National Institutes of Health Baltimore Maryland USA; ^3^ Department of Developmental and Molecular Biology Albert Einstein College of Medicine Bronx New York USA; ^4^ Institute for Aging Research Albert Einstein College of Medicine Bronx New York USA; ^5^ Department of Medicine, Marion Bessin Liver Research Center Albert Einstein College of Medicine Bronx New York USA

**Keywords:** autophagy, CD4^+^ T cells, healthy aging

## Abstract

Autophagy is intricately linked with protective cellular processes, including mitochondrial function, proteostasis, and cellular senescence. Animal studies have indicated that autophagy becomes dysfunctional with aging and may contribute to T cell immunosenescence. In humans, it remains unclear whether autophagy is impaired in CD4^+^ T cells as people age. To answer this question, we examined basal and inducible autophagic activity in a series of experiments comparing CD4^+^ T cells from younger (23–35 years old) and older (67–93 years old) healthy donors. We used immunofluorescence to detect LC3 (a marker of autophagosomes and autolysosomes) and LAMP2 (a marker of endolysosomes) in conjunction with bafilomycin A_1_ (which inhibits the acidification of lysosomes) and CCCP (a mitochondrial uncoupler) to manipulate autophagic flux. We found a significantly higher autophagy flux in CD4^+^ T cells from older compared to younger donors and a higher number of LC3^+^ compartments among older donors. Since the overall amount of autophagosomes degraded was comparable between the two groups, we concluded that autophagosome biogenesis was reduced in the older group. Rather than a decline, our findings in healthy older donors point toward a compensatory enhancement of human CD4^+^ T cell autophagy with age, which may be a mechanism behind healthy aging.

## Graphical abstract

Using CD4^+^ T cells from pairs of young and old healthy human donors, we found that autophagy in the older donor cells did not decline with aging suggesting a compensatory enhanced autophagic efficiency of human CD4^+^ T cells with age, which may be an adaptive mechanism behind healthy aging.

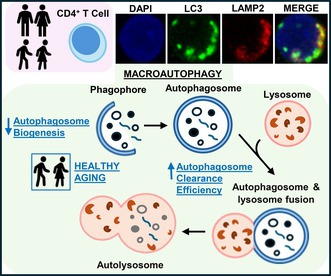

**Abbreviations**
CCCP: carbonyl cyanide *m*‐chlorophenylhydrazone
LAMP2: lysosome‐associated membrane protein 2
LC3: microtubule‐associated protein 1A/1B‐light chain 3
PBMCs: peripheral blood mononuclear cells

## 1 Introduction

Aging is often described as a progressive decline in biological resilience, reducing the ability to repair molecular and cellular damage caused by environmental stress and entropy. The resulting accumulation of damage impairs multiple physiological systems, including the immune system. In particular, T cell dysfunction has been linked to age‐related disorders such as Alzheimer's disease, cardiovascular disease, diabetes, infections, autoimmunity, cancer, and increased mortality (Bektas et al. 2017).

Canonical autophagy is a key homeostatic process where cells degrade and recycle cytoplasmic components like proteins, organelles, and pathogens by sequestering them in autophagosomes and then fuse with lysosomes for breakdown and reuse (Aman et al. 2021). Proper autophagy is critical for proteostasis, mitochondrial integrity, and the controlled regulation of cellular senescence. Hence, the impairment of autophagy can impact immunosenescence through multiple downstream effects (Mittelbrunn and Kroemer 2021). This becomes particularly important at older age, as the burden of damage to macromolecules and organelles becomes higher.

Previous studies suggested that in older individuals, CD4^+^ T lymphocytes have impaired mitochondrial health due to inefficient macroautophagy, specifically mitophagy—the selective removal of damaged mitochondria, which allows the generation of new ones (Bektas et al. 2019; Picca et al. 2023).

Studies, largely in animal models, indicate that autophagy becomes dysfunctional with aging and such decline is thought to contribute to T cell immunosenescence (Macian 2019). Conversely, individuals with exceptional longevity and their cohort display more robust induction of autophagy (Raz et al. 2017), but changes in autophagy with aging in healthy individuals have not been directly evaluated.

Previous research has shown impaired autophagy in senescent CD8^+^ T cells from healthy individuals, which correlates with higher DNA damage (Phadwal et al. 2012). The autophagy‐promoting metabolite spermidine decreases with age in human CD8^+^ T cells, and spermidine supplementation restores autophagy in activated CD8^+^ T cells (Alsaleh et al. 2020). However, these findings may not apply directly to CD4^+^ T cells, as metabolic, phenotypic, and functional changes with aging are substantially different in CD4^+^ and CD8^+^ T cells. For instance, while the relative proportions of memory subsets in CD4^+^ T cells remain relatively stable with age, the central memory compartment of CD8^+^ T cells shrinks by up to 50%, with a corresponding rise in terminally differentiated effector cells (Almanzar et al. 2005; Czesnikiewicz‐Guzik et al. 2008; Zhang et al. 2016). Despite the wealth of omics (transcriptomics and proteomics) reporting expression of autophagy components in multiple human cells, including CD4^+^, the functional status of autophagy can only be determined by directly analyzing the biogenesis and degradation of autophagic compartments, what has been described as autophagic flux (Klionsky, Abdel‐Aziz, et al. 2021), since, for example, an increase in steady‐state levels of LC3‐II, a key protein for macroautophagy, can be a result of both increased autophagic activity or decreased degradation of autophagic compartments. Limited research has explored whether autophagy declines in human CD4^+^ T cells with age, especially in people who stay healthy into old age. For example, a study of patients with rheumatoid arthritis found that CD4^+^ T cells, compared to controls, lacked 6‐phosphofructo‐2‐kinase/fructose‐2,6‐bisphosphatase 3 (F2,6BP)—an enzyme crucial for glycolysis. This deficiency prevented CD4^+^ T cells from using autophagy as an alternative means to provide precursor molecules for energetic metabolism and biosynthetic processes (Yang et al. 2013). Another study found that CD4^+^ T cells from offspring of long‐lived parents exhibited better autophagic activity than those from age‐matched controls (Raz et al. 2017). Notably, there have been no direct studies comparing autophagy in CD4^+^ T cells between healthy young and older individuals, leaving the question of whether autophagy decreases in CD4^+^ T cells with age unresolved. Addressing this question is important because autophagy is vital for maintaining lymphocyte function, and defects in autophagy may contribute to immunosenescence (Macian 2019).

In this study, we investigated autophagy in CD4^+^ T cells from younger and older individuals who were free of chronic diseases. Based on prior research in mice, we hypothesized that autophagy would be lower in CD4^+^ T cells from older participants compared to younger ones. Conversely, our findings indicate that autophagy in CD4^+^ T cells from healthy donors is preserved with aging, maintained by increased autophagic efficiency. This observation points to adaptive mechanisms that should be explored in future studies.

## 2 Results

### 2.1 Basal Autophagic Activity

**FIGURE 1:**
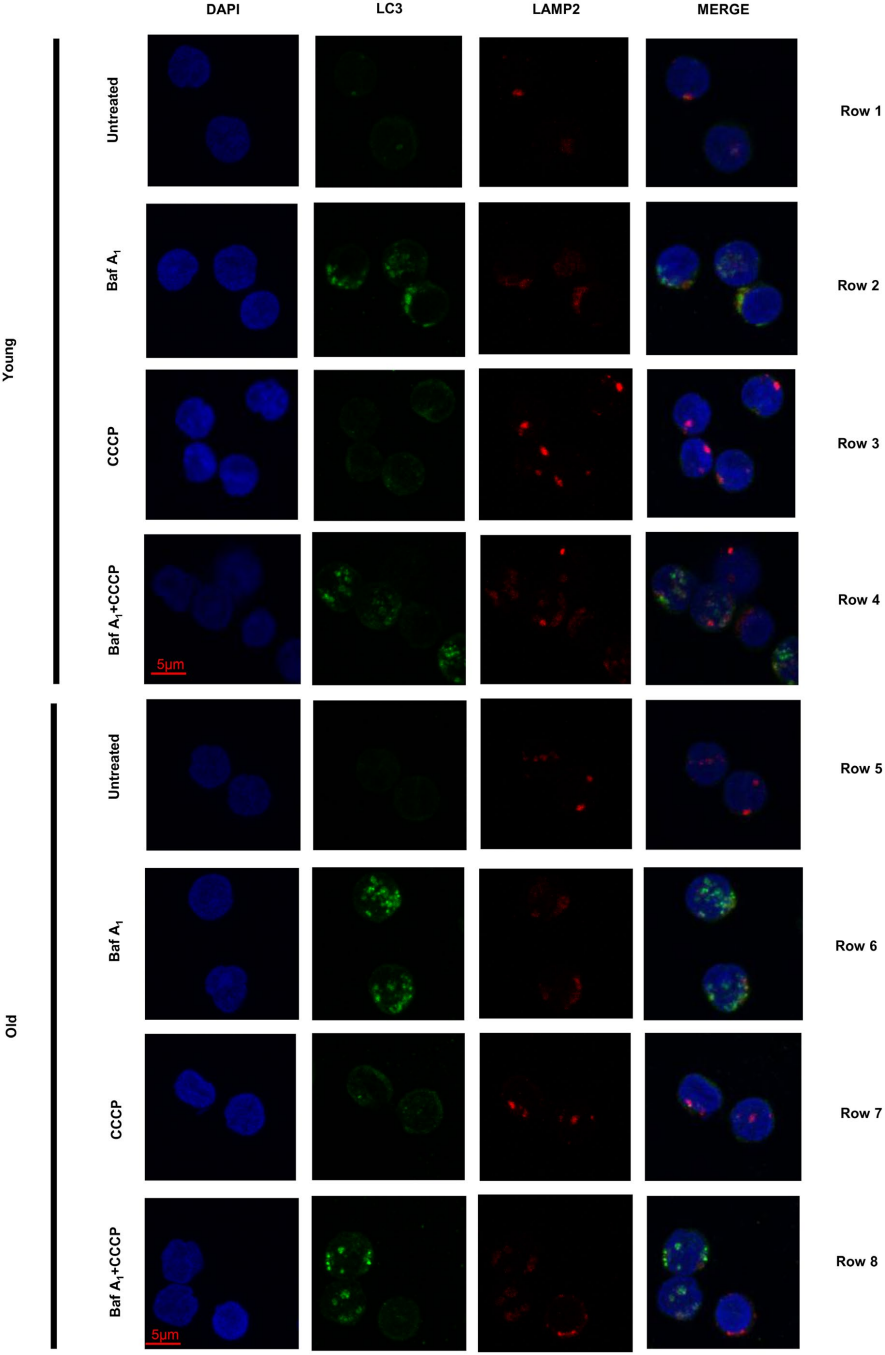
Representative images of untreated, bafilomycin A_1_‐treated, CCCP‐treated, and bafilomycin A_1_ + CCCP‐treated CD4^+^ T cells from young and old donors (rows) that are immunofluorescence‐stained for DAPI, LC3, LAMP2, and merged (columns).

**FIGURE 2:**
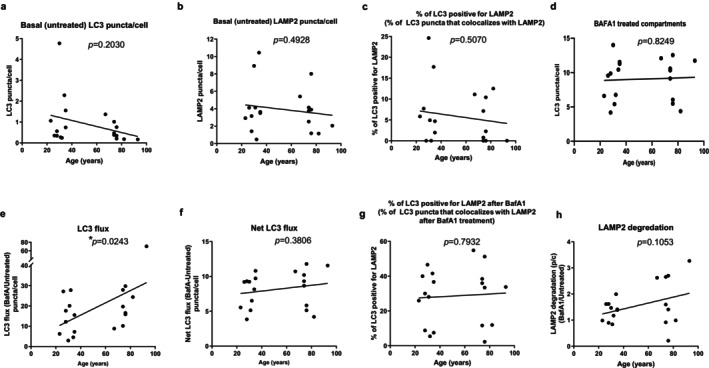
Basal autophagic activity in CD4^+^ T cells from young and old healthy donors. (a) Analysis of LC3‐positive compartments (autophagosomes and autolysosomes) for puncta per cell in untreated cells. (b) Analysis of LAMP2‐positive compartments (endolysosomes), puncta per cell in untreated cells. (c) Analysis of colocalized LC3 and LAMP2 compartment (autolysosomes) in untreated cells. (d) Analysis of LC3‐positive compartments (autophagosomes and autolysosomes) for puncta per cell in bafilomycin A_1_‐treated cells. (e) Analysis of LC3 flux (autophagy flux), expressed as the ratio of LC3 puncta per cell after bafilomycin A_1_ treatment to before (untreated). (f) Analysis of net LC3 flux, expressed as the difference between LC3 puncta per cell of bafilomycin A_1_‐treated cells and untreated cells. (g) Analysis of co‐localized LC3‐LAMP2 puncta per cell (autolysosomes) showing maturation of autophagosomes into autolysosomes in the two age groups. (h) Analysis of LAMP2 degradation expressed as the ratio of LAMP2 puncta per cell after bafilomycin A_1_ treatment to before (untreated). The number of independent experiments is nine, with each experiment containing one young and one old donor, with the exception of experiment 7, which has two young donors and one old donor. *p* values were calculated using a likelihood ratio test to compare this model against a null model without the age variable using ANOVA. *p < 0.05.

To analyze the basal state of autophagy in CD4^+^ T cells from young and old healthy donors, we performed immunostaining and assessed the number of microtubule‐associated protein 1A/1B‐light chain 3 (LC3)‐positive compartments (autophagosomes and autolysosomes). In the steady state condition, the basal number of LC3^+^ puncta tended to be lower in cells from older (age range 67–93 years) than in younger (age range 28–35 years) donors, although the difference was not statistically significant (age_coeff = −0.0157, age_*p* = 0.2030) (representative images in Figure 1, rows 1 and 5; and data in Figure 2a for puncta/cell). Reduction in the number of puncta did not seem attributable to higher clustering of these compartments in the old donors' cells, as we observed a similar trend when considering the fraction of cellular area occupied by LC3^+^ compartments (Figure S1a shows quantification of LC3^+^ area per cell). Remarkably, this trend of lower basal LC3 in CD4^+^ T cells from older donors was consistently found in eight of nine experimental pairs of donors (age_coeff = −0.0157, age_*p* = 0.2030; *p* = 0.0196 for a two‐sided sign test for 8/9 experiments with lower basal CD4^+^ T cells from older as compared to cells from younger donors, Figure S1b for puncta/cell).

In contrast, the average number of endolysosomes, indicated by lysosome‐associated membrane protein 2 (LAMP2)‐positive compartments, was similar in both age groups (age_coeff = −0.0169, age_*p* = 0.4928; see representative images in Figure 1, rows 1 and 5, and puncta per cell in Figure 2b). Additionally, the percentage of total LC3^+^ vesicles (autophagic vacuoles) that were autolysosomes—defined as compartments with colocalized LC3 and LAMP2—was also comparable between the two age groups (age_coeff = −0.0424, age_*p* = 0.5070; Figure 2c).

To understand whether the baseline lower number of LC3 puncta in the older group could be a result of reduced autophagosome biogenesis (lower autophagy induction) or increased autophagosome clearance (degradation) or both, we next analyzed changes of LC3 puncta/cell number and area/cell following treatment with bafilomycin A_1_. Bafilomycin A_1_ inhibits proton pumps in lysosomes, preventing the acidification necessary for lysosomal enzyme activity, therefore leading to the accumulation of LC3 puncta. After bafilomycin A_1_ treatment, we found no significant difference (age_coeff = 0.0062, age_*p* = 0.8249) in LC3 puncta/cell between CD4^+^ T cells from younger and older individuals (representative images in Figure 1, rows 2 and 6, and shown in Figure 2d for puncta/cell and Figure S1c for area/cell). Figure S1d shows individual values within each experiment for basal (untreated) and bafilomycin A_1_‐treated compartments. The ratio of LC3 puncta or LC3 area per cell before and after treatment (autophagy flux, representative images in Figure 1, rows 1, 2, 5, and 6; and shown in Figure 2e for puncta/cell and Figure S1e for area/cell) shows a significantly higher flux (*p* < 0.05) in CD4^+^ T cells from older as compared to younger donors for puncta/cell (age_coeff = 0.3547, age_*p* = 0.0243) as well as for area/cell (age_coeff = 0.4602, age_*p* = 0.0406). Supporting the robustness of these findings, in all eight of the experiments conducted on cells collected from a pair of older and younger individuals, the autophagy flux was higher in the cells from older than younger individuals (comparison illustrated in individual experiment values for bafilomycin A_1_/basal (none) and LC3 puncta/cell in Figure S1f). Only one experiment, in which an older individual was compared to two younger individuals, failed to yield this result (for 8/9 experiments with autophagy flux higher in CD4^+^ T cells from older as compared to younger donors *p* = 0.0196 for a two‐sided sign test). Since the ratio of LC3^+^ puncta in cells treated or not with bafilomycin A_1_ informs mainly on the efficiency of the system (effective flux), but not necessarily on possible changes in the amount of autophagic compartments degraded in each condition, we also calculated the net flux as the difference of LC3 puncta/cell after and before bafilomycin A_1_. We found that net flux was not significantly different between CD4^+^ T cells from older compared to younger participants when considering the overall number of puncta (age_coeff = 0.0207, age_*p* = 0.3806) (Figure 2f) or when analyzing bafilomycin A_1_‐induced changes in LC3^+^ area/cell (age_coeff = 0.0082, age_*p* = 0.6762) (Figure S1g). These findings indicate that, although autophagosomes are more effectively broken down by lysosomes in CD4^+^ T cells from the older group (demonstrated by higher LC3 flux), the total amount of autophagosomes degraded over the same time period was similar (showing a comparable net LC3 flux). Further supporting these results, the older group displayed a lower steady‐state number of LC3 puncta. These findings are consistent with lower autophagosome biogenesis in the older group, but no deterioration of lysosomal degradation with older age. Consistent with this idea, we found a comparable fraction of co‐localized LC3‐LAMP2 puncta/cell (autolysosomes) in the older compared to the young group, again indicative of a similar efficient maturation of autophagosomes into autolysosomes in the two age groups (age_coeff = 0.0397, age_*p* = 0.7932) (Figure 2g).

As a side observation, we took advantage of the bafilomycin A_1_ treatment to also interrogate the stability of the lysosomal compartment, since the integrity of the lysosomal membrane has been shown to decrease in some organisms with age. LAMP2 is an integral lysosomal component with a long half‐life (> 2 days), and as such, its degradation should be unnoticeable during the time of our experiment, unless there is lysosomal breakage and subsequent release of lysosomal enzymes. As expected, degradation of LAMP2 was negligible in CD4^+^ T cells from the younger group (Figure 2h shows no change in the number of LAMP2 puncta upon bafilomycin A_1_ treatment in the younger group illustrated by the ~1 ratio of the number of LAMP2 puncta following bafilomycin A_1_ treatment compared to untreated). In the older donor group, although overall LAMP2 degradation was not significantly different from the younger group (age_coeff = 0.0113, age_*p* = 0.1053), we found higher heterogeneity, with four out of the nine samples displaying increased LAMP2 degradation (~3 ratio of the number of LAMP2 puncta following bafilomycin A_1_ treatment compared to untreated, Figure 2h). Interestingly, despite some possible reduction in lysosomal stability with age in CD4^+^ T cells, their autophagic efficiency (ability to degrade autophagosomes) was still well preserved.

We performed sensitivity analyses to determine if (1) removal of experiment 1, which included the analysis of data from a 93 year‐old‐man as an outlier, would result in meaningful change in the results for inducible autophagic flux following bafilomycin A_1_ treatment and (2) if removal of experiments 1 and 7, which included data from both men and women within the same experiment, would result in meaningful change in the inducible autophagic flux results. Removal of experiment 1 showed that, though while no longer significant by linear mixed effect analysis, autophagic flux in the older group remained higher compared to the younger group (age_coeff = 0.1295, age_*p* = 0.1897 for puncta/cell, Figure S1h, left, and age_coeff = 0.2059, age_*p* = 0.2550 for area/cell, Figure S1h, right). Consistency of the effect by age in seven out of eight experiments remained statistically significant (sign test: *p* = 0.0339). After removing experiments that included a man and woman (experiments 1 and 7), the higher autophagy flux in the older group compared to the younger group was still statistically significant (age_coeff = 0.2171, age_*p* = 0.0320 for puncta/cell, Figure S1i, left, and for area/cell, age_coeff = 0.3787, age_*p* = 0.0397, Figure S1i, right).

### 2.2 Inducible Autophagic Activity

**FIGURE 3:**
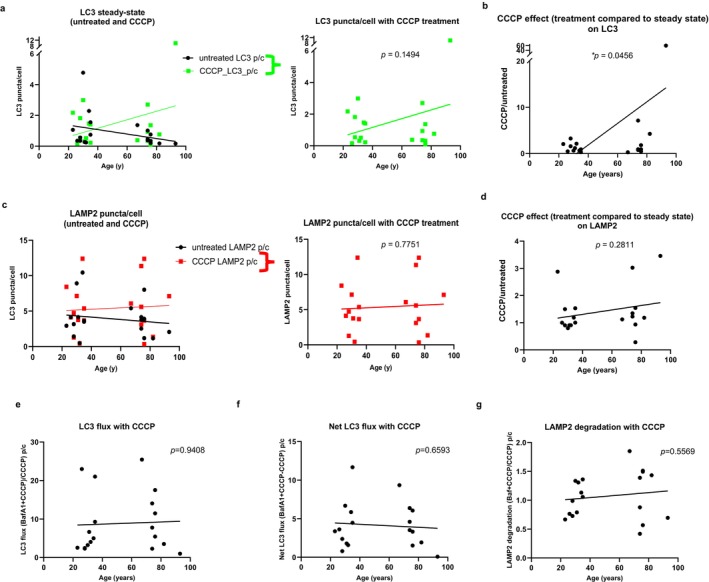
Inducible autophagic activity and discrimination between higher induction of autophagy or reduced autophagosome clearance in CD4^+^ T cells from young and old healthy donors. (a) Left, analysis of LC3 positive compartments (autophagosomes and autolysosomes) for puncta per cell in untreated cells (black, basal repeated from Figure 1a) and CCCP‐treated cells (green). Right, CCCP‐treated cells (green) graph alone. (b) Analysis of CCCP effect, expressed as the ratio of LC3 puncta per cell after CCCP treatment to before (untreated). (c) Left, analysis of LAMP2‐positive compartments (endolysosomes) for puncta per cell in untreated cells (black, basal repeated from Figure 1c) and CCCP‐treated cells (green). Right, CCCP‐treated cells (green) graph alone. (d) Analysis of CCCP effect, expressed as the ratio of LAMP2 puncta per cell after CCCP treatment to before (untreated). The number of independent experiments is nine, with each experiment containing one young and one old donor, with the exception of experiment 7, which has two young donors and one old donor. *p* values were calculated using a likelihood ratio test to compare this model against a null model without the age variable using ANOVA. (e) Analysis of LC3 flux (autophagy flux), expressed as the ratio of LC3 puncta per cell after bafilomycin A_1_ + CCCP treatment to CCCP treatment. (f) Analysis of net LC3 flux, expressed as the difference between LC3 puncta per cell of bafilomycin A_1_ + CCCP‐treated cells and CCCP‐treated cells. (g) Analysis of LAMP2 degradation, expressed as the ratio of LAMP2 puncta per cell after bafilomycin A_1_ + CCCP treatment to CCCP treatment. The number of independent experiments is nine, with each experiment containing one young and one old donor, with the exception of experiment 7, which has two young donors and one old donor. *p* values were calculated using a likelihood ratio test to compare this model against a null model without the age variable using ANOVA. *p < 0.05.

The considerable robustness of the basal autophagic/lysosomal function observed in CD4^+^ T cells from both younger and older donors motivated us to study how the autophagic system responds to stress at different ages. To that end, we used the mitochondrial uncoupler carbonyl cyanide *m*‐chlorophenylhydrazone (CCCP) that, by inducing an energetic stress, stimulates autophagy [preferentially mitophagy (Bektas et al. 2019)]. Addition of CCCP resulted in an overall increase in the number of LC3‐positive compartments from steady state in the older age group that was not observed in the younger one, although the difference was not statistically significant (age_coeff = 0.0364, age_*p* = 0.1494); representative images in Figure 1, rows 3 and 7; and LC3 puncta/cell in Figure 3a. The previously observed lower number of LC3‐positive puncta in CD4^+^ T cells from old compared to CD4^+^ T cells from younger donors (Figure 2a) was no longer noticeable. Analysis of the effect of CCCP on LC3 in each age group revealed significantly higher increases in the number of LC3 puncta in the older group (age_coeff = 0.3060, age_*p* = 0.0456) (Figure 3b; Figure S2). We did not observe substantial changes in the number of endolysosomal compartments (LAMP2 positive) upon addition of CCCP (age_coeff = 0.0099, age_*p* = 0.7751; Figure 3c), except from two old and one young donor samples that showed a 2‐fold increase (age_coeff = 0.0081, age_*p* = 0.2811) (Figure 3d).

**FIGURE 4:**
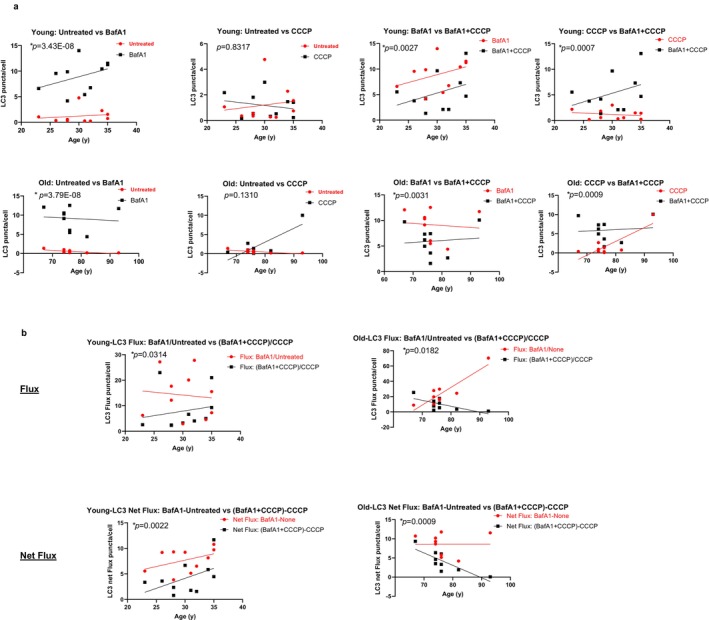
Comparing young and young, and old and old before and after treatments in CD4^+^ T cells from healthy donors. (a) Top row, young to young comparisons for (from left to right) bafilomycin A_1_‐treated versus untreated, CCCP‐treated versus untreated, bafilomycin A_1_ + CCCP‐treated versus bafilomycin A_1_‐treated, and bafilomycin A_1_ + CCCP‐treated versus CCCP‐treated. Bottom row, old to old comparisons for (from left to right) bafilomycin A_1_‐treated versus untreated, CCCP‐treated versus untreated, bafilomycin A_1_ + CCCP‐treated versus bafilomycin A_1_‐treated, and bafilomycin A_1_ + CCCP‐treated versus CCCP‐treated. (b) Flux (upper row), left, young to young comparison of LC3 flux, expressed as the ratio of LC3 puncta per cell (red) after bafilomycin A_1_ treatment to before (untreated) compared to the LC3 flux expressed as the ratio of LC3 puncta per cell (black) after bafilomycin A_1_ + CCCP treatment to CCCP treatment. Right, old to old comparison of LC3 flux, expressed as the ratio of LC3 puncta per cell (red) after bafilomycin A_1_ treatment to before (untreated) compared to the LC3 flux expressed as the ratio of LC3 puncta per cell (black) after bafilomycin A_1_ + CCCP treatment to CCCP treatment. Net Flux (lower row), left young to young comparison of net LC3 flux, expressed as the difference between LC3 puncta per cell (red) of bafilomycin A_1_‐treated cells and untreated cells compared to the net LC3 flux expressed as the difference between LC3 puncta per cell (black) of bafilomycin A_1_ + CCCP‐treated cells and CCCP‐treated cells. Right, old to old comparison of net LC3 flux, expressed as the difference between LC3 puncta per cell (red) of bafilomycin A_1_‐treated cells and untreated cells compared to the net LC3 flux expressed as the difference between LC3 puncta per cell (black) of bafilomycin A_1_ + CCCP‐treated cells and CCCP‐treated cells. The number of independent experiments is nine, with each experiment containing one young and one old donor, with the exception of experiment 7, which has two young donors and one old donor. *p* values were calculated using a likelihood ratio test to compare this model against a null model without the age variable using ANOVA. *p < 0.05.

The relatively higher increase in LC3 compartments observed in the samples from the older donors upon CCCP treatment (Figure 3b) can be the result of either a higher induction of autophagy or a reduced autophagosome clearance. To discriminate between these two possibilities, we analyzed autophagic flux upon the addition of CCCP to bafilomycin A_1_‐treated cells (representative images in Figure 1, rows 3, 4, 7, and 8). Contrary to preclinical studies reported in the literature (Kane et al. 2018), CCCP addition did not consistently increase the autophagic flux in all donor samples regardless of age (age_coeff = 0.0063, age_*p* = 0.9408 comparing old to young in Figure 3e; and in Figure 4a comparing young to young and old to old after adding CCCP to bafilomycin A_1_). In fact, autophagic flux and net autophagic flux were noticeably lower upon CCCP induction in both age groups (Figure 4b; Figure S3). Figure S3 shows changes in overall LC3 puncta upon the addition of CCCP and/or bafilomycin A_1_ for each individual in the different experiments: only in one sample from a young donor (35 years [Y2] in experiment 5) did we observe a higher LC3 degradation upon CCCP than under basal conditions (bafilomycin A_1_ + CCCP compared to bafilomycin A_1_), whereas all the rest of the samples showed a consistent CCCP‐induced decrease in LC3 degradation. Interestingly, mixed effects analysis revealed that the addition of CCCP completely abolished the higher LC3 flux observed in CD4^+^ T cells from the older group under basal conditions (compare Figure 2e with Figure 3e). Analysis of the net LC3 flux, although always lower upon CCCP treatment, revealed no differences in the overall amount of autophagosomes undergoing lysosomal degradation upon CCCP addition between both age groups (age_coeff = −0.0132, age_*p* = 0.6593) (Figure 3f). The addition of CCCP per se did not aggravate the lysosomal instability (LAMP2 degradation) (age_coeff = 0.0022, age_*p* = 0.5569) (Figure 3g).

Overall, our findings support no major compromise of autophagic activity in CD4^+^ T cells from healthy individuals under basal conditions with age, but a lower ability, albeit discrete, to maintain autophagic activity in response to the CCCP stressor.

## 3 Discussion

Many studies, mostly in animals, indicate that autophagy becomes less effective as organisms age. Studies in animal models such as *Caenorhabditis elegans* , rodents, and in some human cells have shown an age‐dependent decrease in proteolytic lysosomal function, which impairs autophagy flux and appears to be associated with age‐related disorders (Aman et al. 2021). The hypothesized role of autophagy in the aging process has also been supported by pharmacological and genetic manipulations of autophagy‐related genes that show tissue‐specific functional changes and/or concordant lengthening or shortening of longevity in experimental animal models (Aman et al. 2021).

**FIGURE 5:**
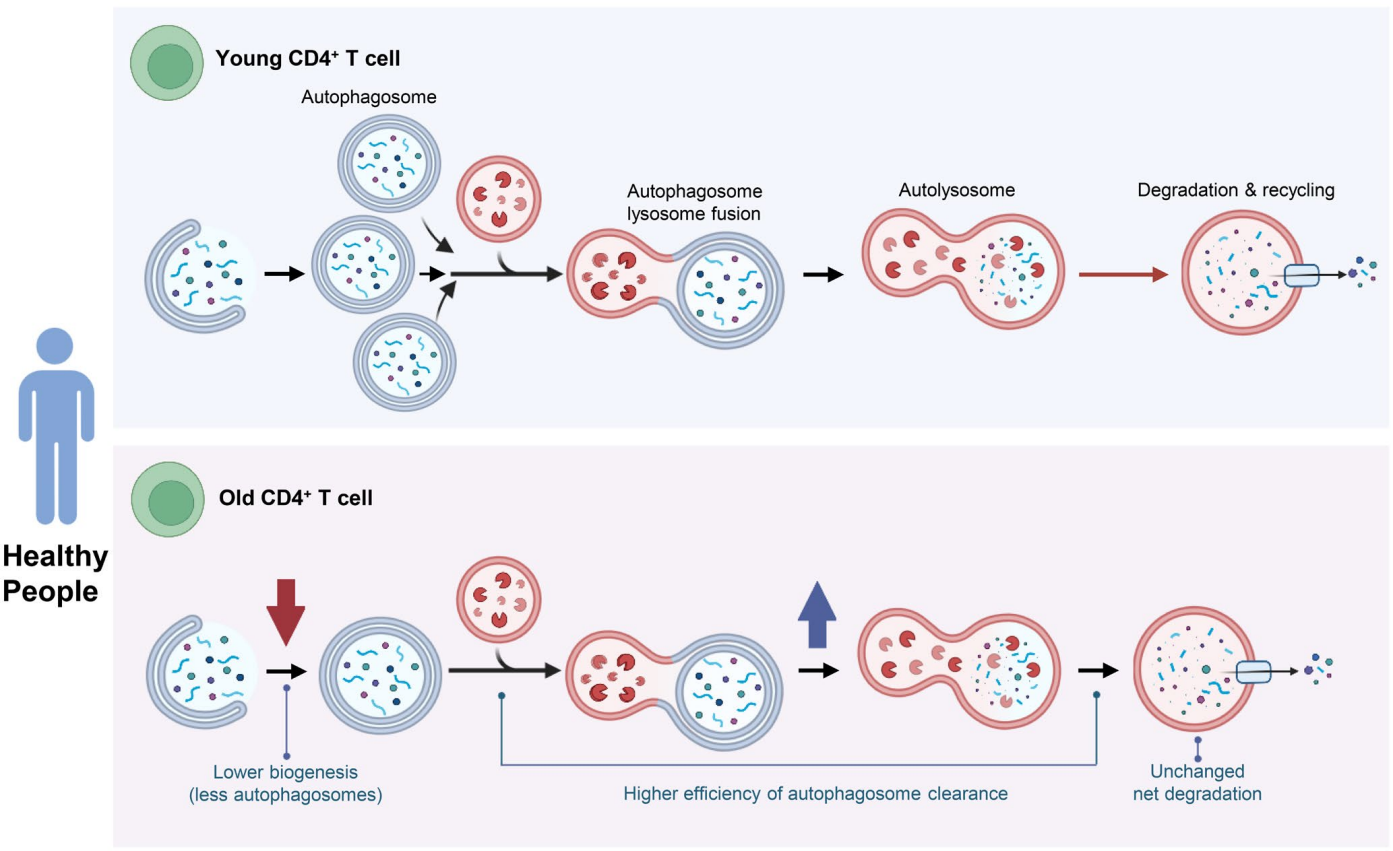
Hypothetical model of adaptive changes in the autophagic/lysosomal system of CD4^+^ T cells in old healthy individuals. In healthy cells, autophagy starts with the formation of a double‐membrane structure called autophagosome, which encloses the cellular material to be degraded. This autophagosome fuses with a lysosome, forming an autolysosome, where the sequestered contents are degraded and recycled to maintain cellular homeostasis. In our model, we suggest that CD4^+^ T cells from healthy old individuals show reduced autophagosome biogenesis and higher efficiency of fusion/degradation by lysosomes when compared to young ones (depicted by a red longer arrow to illustrate longer time to complete degradation). We propose that the decrease in autophagosome biogenesis with age is probably a compensatory process to reduce cargo delivery to lysosomes that may not have been as functional as in the young individuals. This ability for healthy elderly individuals to maintain effective autophagic processes in CD4^+^ T cells may help these cells succeed in eliciting an accommodating mechanism that will preserve functionality longer. This figure was created with the aid of BioRender.

It is not yet clear whether the same decline in autophagy happens in aging humans, particularly those who remain healthy and relatively active in their later years. In this study involving healthy older adults, we assessed autophagy parameters in CD4^+^ T cells collected from pairs of healthy younger and older individuals. Contrary to our hypothesis, we found that in CD4^+^ T cells from healthy donors, autophagy does not decline with aging and shows some signs of compensatory enhancement. Surprisingly, the efficiency with which these cells clear autophagosomes was significantly higher in older donors compared to younger ones. Interestingly, while the rate of autophagosome clearance was higher in samples from older donors, the overall crude amount of autophagosomes degraded was comparable (Figure 5). Since these changes were not associated with changes in the percentage of autophagic vacuoles that had matured into autolysosomes at a given time, we conclude that autophagosome biogenesis is reduced in the older group (Figure 5). This finding could be interpreted as specific compensatory mechanisms to maintain autophagy, although at least theoretically such a strategy could make older persons more susceptible to overt stress challenges that require full functional autophagy. While previous research pointed to a decline in autophagy with age, we found that healthy elderly individuals maintain effective autophagic processes in CD4^+^ T cells. The observed compensatory mechanisms that avoid the buildup of autophagosomes are similar to what has been previously described in some rodent organs with age (Park et al. 2022; Stavoe et al. 2019; Tsong et al. 2023).

Our study did not find overt differences in lysosomal stability between CD4^+^ T cells from younger and older donors, but there was a trend toward aberrant LAMP2 degradation in cells from older donors. This, along with possible yet‐to‐be‐explored differences in lysosomal pH or enzymatic content, could trigger the observed reduction in autophagosome biogenesis in order to reduce the amount of cargo delivery to less functional lysosomes. It is anticipated that cells that succeed in eliciting this accommodating mechanism will preserve functionality longer. A burning question is whether the preservation of effective autophagy in these healthy individuals free of chronic diseases is one of the reasons for the maintenance of their healthy status. This hypothesis should be tested in future studies that compare the status of autophagy and the endolysosomal system between healthy and frail individuals in the same age range.

Our original intention when introducing CCCP in the study design was to compare basal and inducible autophagy, since CCCP has proven effective in upregulating autophagy in different experimental models. Unexpectedly, we found that CCCP had an inhibitory effect on the autophagic flux in CD4^+^ T cells from both young and old individuals. It is possible that concentrations optimized for use in experimental animal models cause excessive mitochondria depolarization in human cells, leading to energetically compromised cells unable to sustain proper autophagic function. In addition, although CCCP normally has been reported to induce autophagy, it has also been reported to inhibit autophagy under certain conditions (Klionsky, Abdel‐Aziz, et al. 2021; Padman et al. 2013). We used data collected after CCCP exposure to analyze the robustness of the autophagic system and ability to respond to this stress. The CCCP challenge revealed a slightly higher vulnerability of the autophagic lysosomal system in the older group compared to the younger group. The initial lower amount of autophagosomes detected in the older group was no longer evident after CCCP, possibly because of lower net autophagosome degradation in cells from older participants.

Earlier work in rodents and other animal models has shown that autophagy declines with aging. However, more recently, Yamamuro et al. (2020) found that there was an age‐related increase in autophagy flux in murine adipocytes. Also, Bensalem et al. (2023) found that autophagy flux in peripheral blood mononuclear cells (PBMCs) of diabetic individuals increased with age. Recent evidence supports the premise that attention to cell and tissue specificity and disease status in studying autophagy in humans is important, and caution should be taken when examining autophagy in animal models as surrogates for studying human autophagic processes, in particular those related to age.

There is evidence in the literature for clear species differences, and selective pressures may influence the preservation of autophagy efficiency in long‐lived species. Individuals with defective autophagy may experience premature mortality or be affected by chronic diseases that would have prevented them from participating in our study. In fact, dysregulation of autophagy has been found, primarily through murine models, to be related to many diseases/disorders seen in humans including neurodegenerative, cardiovascular, pulmonary, hepatic, renal, and neoplastic diseases. Specific disease relationships with autophagy perturbations have been extensively reviewed in Klionsky, Petroni, et al. (2021). Conversely, pharmacological treatments, including spermidine, resveratrol, Urolithin A, and tomatidine in animal models such as *C. elegans* , *Drosophila*, and mice have been shown to extend animal lifespan and healthspan through the activation of autophagy (Nakamura and Yoshimori 2018).

From an evolutionary perspective, bats show evidence of increased autophagy with age compared to other mammals. In an 8‐year longitudinal analysis, Huang et al. (2019) showed using transcriptomics analysis that a long‐lived bat species exhibited upregulation of the autophagic transcriptional program with advancing age. Bats are the longest‐lived mammals for their size, and the group found that 10 autophagy‐associated genes were under selected pressure in bat lineages (Kacprzyk et al. 2021). Bats have overall the greatest longevity quotient [observed/expected longevity of 352 analyzed mammalian species (Locatelli and Cenci 2022)] and are similar to another long‐lived species for its size, the naked mole rat, where preserved autophagic activity has also been reported with age.

Our findings indicate that among participants screened as healthy, autophagy flux increases with age in CD4^+^ T‐cells. This may suggest that autophagic processes are adapting over time to support survival in long‐lived mammals, especially in those who remain healthy up to late in life. As damage accumulates with aging, the body may need to enhance autophagy to cope with this increased stress. Thus, our finding of lower autophagy at baseline in spite of damage accumulation may in itself be a sign of a deficit. When autophagy is stimulated, the greater increase in older adults may be the result of a much larger amount of damaged material that needs to be processed. Thus, as more damage accumulates in older age, one may expect that the autophagy flux needed to maintain homeostasis needs to be higher. The older individuals studied here may still be healthy because of their conserved autophagy, and therefore autophagy flux could potentially be developed into a biomarker of healthy aging or, in the specific context of CD4^+^ T cells, a biomarker of immune fitness.

Since it is likely that molecular damage accumulation in cells from older persons is higher than in cells from younger persons, one could speculate that enhancement of autophagosome biogenesis could be beneficial to maintain a healthy status, as far as it is accompanied by an improvement of the lysosomal system, which ultimately will be responsible for degrading those autophagosomes. Chang et al. (2021) recently outlined the mechanisms involved in autophagosome biogenesis and function. Key among the autophagy core complexes and possible targets of manipulation to improve autophagosome biogenesis include the ULK1 complex (activated by the mechanistic target of rapamycin inhibition and also through starvation, hypoxia, ER stress), the class III phosphatidylinositol 3‐kinase (PI3K‐III) complex, and the BECN1/Beclin 1 (regulatory subunit stimulated by ER stress, hypoxia, microbial infection) (Chang et al. 2021; Nakatogawa 2020).

In particular, BECN1 is a core component of the PI3K‐III complex and, through modulation of the lipid kinase activity of the PI3K‐III catalytic unit VPS34, which generates phosphatidylinositol 3‐phosphate [PI(3)P], allows the recruitment of a number of autophagy proteins involved in the nucleation of the autophagosome (McKnight and Zhenyu 2013). In mice, the overexpression of Beclin 1 has been shown to stimulate autophagy and has also been shown to be protective of several disease models such as spinocerebellar ataxia type 3 (Nascimento‐Ferreira et al. 2011). A derivative of BECN1, Tat‐BECN1 (He et al. 2022), has demonstrated autophagy induction in the heart, skeletal muscle, and pancreas after being administered intraperitoneally into mice (Shoji‐Kawata et al. 2013). A myriad of mouse cells, including CD8^+^ T cells and many human cell types, including CD4^+^ T cells (Zhang et al. 2019), have been shown to be responsive to Tat‐BECN1/Beclin 1 (He et al. 2022). In fact, a mutation in *Becn1* that disrupts the interaction of Beclin 1 with the negative regulator BCL‐2 extends both the healthspan and lifespan in mice and has increased basal autophagy (Fernandez et al. 2018).

However, any attempt to increase autophagosome biogenesis, to be successful, should be accompanied by efforts to improve the quality/function of the lysosomal system. In that respect, interventions proven to increase both autophagosome and lysosomal biogenesis, such as the activation of the transcription factor TFEB, may be proven effective in preserving autophagic flux.

Our results also point toward the possible health‐preserving benefits of increased autophagy and autophagy flux. Many studies in mice and rats and some human studies have shown that caloric restriction increases autophagy and autophagy flux in organs and tissues such as the liver, muscle, adipose, and kidney (Chung and Chung 2019). Caloric restriction mimetics (CRMs) such as metformin, an AMPK activator, rapamycin, an mTOR inhibitor, and spermidine, acting through inhibition of acetyltransferases, each are known to increase autophagy and have effects on immune resilience. Metformin has been shown to affect the differentiation and activation of immune‐mediated cells such as CD4^+^ and CD8^+^ T cells (Nojima and Wada 2023) and has been shown to ameliorate a Th17 inflammaging profile by enhancing autophagy and normalizing mitochondrial function (Bharath et al. 2020). Regarding rapamycin‐related immune effects, a randomized placebo‐controlled phase 2a clinical trial did show that a low dose combination of a catalytic (BEZ235) plus an allosteric (RAD0001) mTOR inhibitor that selectively inhibits TORC1 had a significant decrease in the rate of reported infections after 1 year (Mannick et al. 2018). Spermidine has been shown to have multiple effects on immune cells able to reverse immune senescence through the translational control of autophagy, though most often through effects on lysosomes and through TFEB expression, which preferentially affects lysosomal biogenesis (Zhang and Simon 2020).

These adaptive mechanisms for autophagy need further study in both healthy and unhealthy individuals across the healthspan and lifespan.

## 4 Methods

### 4.1 Human Samples

Samples for this study were obtained from healthy participants in the Baltimore Longitudinal Study of Aging (BLSA) and from additional healthy donors (Table S1). This included eight younger individuals (aged 28–35) and six older individuals (aged 67–93). BLSA participants (enrolled under NIA protocol #03‐AG‐0325) volunteered to donate blood via the Tissue Procurement for Biomedical Research protocol (NIA protocol #03‐AG‐N322) or apheresis material via the Cytapheresis of Volunteer Donors protocol (NIA protocol #03‐AG‐N316). Sample collection was approved by the NIH Institutional Review Board, and all participants gave informed consent, acknowledging the potential risks of participation.

### 4.2 Cells and Treatments

In all nine experiments, CD4^+^ T cells from young and old individuals were assessed in the same session. Eight experiments assessed one young and one old individual, while one experiment assessed two young and one old individual. In five of the nine experiments, we intentionally reused cells (not the data) from a previous donor as an internal control (repeated younger donor in experiments 2 and 5; repeated older donor in experiments 7, 8, and 9; see Table S1). This design choice was accounted for in the analysis using a mixed‐effect model.

To obtain CD4^+^ T cells, PBMCs were first isolated from apheresis or whole blood using density gradient centrifugation with Ficoll‐Paque Plus (StemCell Technologies). CD4^+^ T cells were then separated from the freshly isolated PBMCs using an immunomagnetic negative selection cell isolation kit (EasySep Human Cell Enrichment Kit [19052]; StemCell Technologies) and an automated cell separator (RoboSep; StemCell Technologies) following the manufacturer's instructions. The isolated cells were cryopreserved and stored in a nitrogen freezer at approximately −192°C.

For each experiment, CD4^+^ T cells from a young and an old donor (except for experiment 7 with two young donors and one old donor, where three samples were used) were thawed and resuspended in RPMI 1640 complete medium (10% FBS and 1% P/S/G) into separate cell culture flasks. After thawing, the cells were incubated for 2 h at 37°C, then centrifuged at 1400 rpm for 5 min. The supernatant was discarded, and the cells were resuspended in complete medium and counted. Cells from the young and old donors were then split into 0.5 × 10^6^ cells in 0.5 mL aliquots for four different conditions: Control (no treatment); addition of CCCP (10 μM), a mitochondrial uncoupler used in this study to induce mitophagy; addition of bafilomycin A_1_ (100 nM), an inhibitor of lysosomal acidification and consequently of the autophagosome‐lysosome fusion; addition of both CCCP and bafilomycin A_1_ (10 and 100 μM, respectively) to block degradation of cargo upon induction of mitophagy. The cells from both young and old donors were then incubated for 18 h at 37°C under these four conditions.

### 4.3 Immunofluorescence Staining and Fluorescence Microscopy

#### 4.3.1 Centrifugation and Preparation

After treatment, CD4^+^ T cells were centrifuged at 1400 rpm for 5 min at room temperature (RT); the supernatant was discarded, and cells were resuspended in PBS and then applied to glass slides using a cytospin centrifuge (Thermo Scientific‐Cytospin4) at RT.

#### 4.3.2 Fixation and Permeabilization

Cells were fixed with 4% paraformaldehyde in PBS for 10 min at RT. They were then permeabilized with 0.3% Triton X‐100 (Calbiochem; CAS 9005‐64‐5) for 20 min at RT. The microscopy analysis was mostly focused on the detection of LC3, a protein essential for the formation of autophagosomes (as a marker for autophagosomes) and LAMP2, a lysosomal membrane protein (as a marker for endolysosomes).

#### 4.3.3 Blocking and Primary Antibody Incubation

Cells were washed with PBS and blocked for 1 h in blocking buffer (PBS, 0.1% Tween‐20, 10% normal goat serum) at RT, and then incubated for 1 h at RT with anti‐LC3 antibody (MBL, PM036; 1:300) and anti‐LAMP2 antibody (abcam, ab25631; 1:100).

#### 4.3.4 Washing and Secondary Antibody Incubation

Cells were washed three times with wash buffer (PBS, 0.1% Tween‐20) for a total of 30 min and then incubated with Alexa Fluor 488‐conjugated anti‐rabbit (Invitrogen; A11008) and Alexa Fluor 568‐conjugated anti‐mouse (Invitrogen; A21124) secondary antibodies for 1 h at RT.

#### 4.3.5 Final Wash and Mounting

After secondary antibody incubation, cells were washed three more times with wash buffer for a total of 30 min and then mounted on glass coverslips using mounting medium containing DAPI (ProLong Gold; Invitrogen, P36935) for nuclear staining.

#### 4.3.6 Imaging

Images were captured with a confocal fluorescence microscope (Zeiss LSM 880). Each image was acquired as a *Z*‐stack using a 63× oil objective and saved as a gallery; then, using the processing tab, the *Z*‐stack collapsed into a single *xy* image and maximum‐intensity projection (MIP) was obtained.

In total, 568 images, containing 11,734 CD4^+^ T cells, were analyzed from the nine experiments having a total of 19 donor samples (two donors for each experiment except experiment #7, which had three donors). These cells had been divided into four treatment conditions in each experiment (untreated, bafilomycin A_1_‐treated, CCCP‐treated, and bafilomycin A_1_ + CCCP‐treated) with roughly the same number of cells analyzed per condition (2262, 3820, 2664, and 2988 cells, respectively). Hence, on average, per donor per experiment, 119 untreated cells, 201 bafilomycin A_1_‐treated cells, 140 CCCP‐treated cells, and 157 bafilomycin A_1_ + CCCP‐treated cells were analyzed. Colocalization is analyzed through four images of each condition (untreated cells, bafilomycin A_1_‐treated cells, CCCP‐treated cells, and bafilomycin A_1_ + CCCP‐treated cells) for each participant.

### 4.4 Autophagy Analysis and Measurements

#### 4.4.1 Quantification of Puncta

The number of LC3 and LAMP2 puncta, as well as the total cell area occupied by these puncta, was measured using the FISH module of HALO software (v3.3; Indica Labs).

#### 4.4.2 Autophagic Flux Measurement

Autophagic flux (indicating autophagosome formation and lysosomal degradation) was quantified by comparing bafilomycin A_1_‐treated cells to untreated cells. Flux was calculated as the number of LC3 puncta (or the area occupied by LC3 puncta, to account for possible vesicular clustering) in bafilomycin A_1_‐treated cells divided by the corresponding value in untreated cells. To determine net autophagic flux, the number of LC3 puncta in untreated cells was subtracted from the number in bafilomycin A_1_‐treated cells, showing the changes in the overall amount of autophagosomes formed and eliminated through lysosomal degradation. Changes in LAMP2 stability were calculated as the ratio of the number of LAMP2 puncta (or the area occupied by LAMP2 puncta) in bafilomycin A_1_‐treated cells divided by the corresponding value in untreated cells. The CCCP effect was calculated as, for LC3, the ratio of the number of LC3 puncta (or the area occupied by LC3 puncta) in CCCP‐treated cells divided by the corresponding value in untreated cells; and for LAMP2, the ratio of the number of LAMP2 puncta (or the area occupied by LAMP2 puncta) in CCCP‐treated cells divided by the corresponding value in untreated cells.

#### 4.4.3 Colocalization

The percentage of colocalization between LC3 and LAMP2 was calculated for each of the four experimental conditions (No treatment, bafilomycin A_1_, CCCP, and bafilomycin A_1_ + CCCP) to confirm autophagosome and lysosome fusion. Colocalization was performed using the DiAna plugin in Image J software (Schneider et al. 2012).

Data included in the Section 2 figures are in puncta/cell. All area/cell result figures and some additional puncta/cell figures are included in the Supporting Information.

### 4.5 Statistical Analysis

For each flux measurement, a mixed‐effects model was run according to the formula: flux ~ age + (1|donor), that is, using age as a fixed effect and donor ID as a random effect, implemented in a script via the lmer function from the R package lme4 (v. 1.1.33) with the argument REML = FALSE to optimize the log‐likelihood. The statistical significance of age effects was assessed by performing a likelihood ratio test to compare this model against a null model without the age variable using the ANOVA function from the R package stats (v. 4.3.0) (Gałecki and Burzykowski 2013). All graphs were made using GraphPad Prism v. 10.

Sensitivity analyses were performed to determine whether (1) the removal of experiment 1, which included the analysis of data from a 93 year‐old‐man as an outlier, would result in meaningful change in the results, and (2) if the removal of experiments 1 and 7, which included data from both men and women within the same experiment, would result in meaningful change in the results.

Given our experiments paired CD4^+^ T cells from young and old individuals, we tested whether the direction of the association of age and autophagy with flux across experiments was different from what would be expected by chance by using a two‐sided sign test. Specifically, we compared our results with the null hypothesis that positive and negative values would be equally likely to occur (*p* = 0.5). This analysis was conducted using SAS 13.13.

## Author Contributions

A.B. performed and designed the experiments, captured and analyzed microscopic images, analyzed data, designed the figures, and contributed to the manuscript. S.H.S. analyzed microscopic images, analyzed data, contributed to the figures, and drafted the manuscript. J.C. provided linear mixed effect model analysis and contributed to the manuscript's linear mixed effects section. O.S.‐F. analyzed data, provided the ImageJ program, and edited the manuscript. S.K. analyzed data, provided the ImageJ program, and edited the manuscript. A.M.C. provided expertise in aspects of the autophagy framework regarding results and contributed to the manuscript. L.F. conceived and directed the study, contributed to the manuscript, and edited all versions of the manuscript.

## Conflicts of Interest

The authors declare no conflicts of interest.

## Supporting information


**Figure S1:** Additional analysis of basal autophagic activity in CD4^+^ T cells from young and old healthy donors. (a) Analysis of LC3‐positive compartments (autophagosomes and autolysosomes) for area per cell in untreated cells. (b) For each donor within individual experiments, LC3 puncta per cell values of basal (untreated). (c) Analysis of LC3‐positive compartments (autophagosomes and autolysosomes) for area per cell in bafilomycin A_1_‐treated cells. (d) For each donor within individual experiments, LC3 puncta per cell values of basal (untreated) compared to bafilomycin A_1_‐treated compartments. (e) Analysis of LC3 flux, expressed as the ratio of LC3 area per cell before and after bafilomycin A_1_ treatment. f, for each donor within individual experiments, LC3 flux (autophagy flux), expressed as the ratio of LC3 puncta per cell after bafilomycin A_1_ treatment to before (untreated). (g) Analysis of net LC3 flux, expressed as the difference between LC3 area per cell of bafilomycin A_1_‐treated cells and untreated cells. (a–g) The number of independent experiments is nine with each experiment containing one young and one old donor with the exception of experiment 7, which has two young donors and one old donor. *p* values were calculated using a likelihood ratio test to compare this model against a null model without the age variable using ANOVA. *p < 0.05. (h) Mixed effect sensitivity analyses of LC3 flux (autophagy flux), expressed as the ratio of LC3 puncta per cell (left) or LC3 area per cell (right) after bafilomycin A_1_ treatment to before (untreated) with experiment 1 removed (experiment 1 includes the 93 years old male outlier). The number of independent experiments is eight with each experiment containing one young and one old donor with the exception of experiment 7, which has two young donors and one old donor. *p* value was calculated using a likelihood ratio test to compare this model against a null model without the age variable using ANOVA. (i) Mixed effect sensitivity analyses of LC3 flux (autophagy flux), expressed as the ratio of LC3 puncta per cell (left) or LC3 area per cell (right) after bafilomycin A_1_ treatment to before (untreated) with experiments that compared individuals of different sexes (experiments 1 and 7 removed). Number of independent experiments is seven with each experiment containing one young and one old donor. *p* value was calculated using a likelihood ratio test to compare this model against a null model without the age variable using ANOVA. *p < 0.05.
**Figure S2:** Additional analysis of inducible autophagic activity in CD4^+^ T cells from young and old healthy donors. For each donor within individual experiments, analysis of CCCP effect, expressed as the ratio of LC3 puncta per cell after CCCP treatment to before (untreated).
**Figure S3:** Changes in overall LC3 puncta upon addition of CCCP and/or bafilomycin A_1_ for each individual in the different experiments. For each donor within individual experiments, LC3 puncta per cell values of untreated, CCCP‐treated, and showing LC3 degradation upon CCCP treatment compared to basal conditions (bafilomycin A_1_ + CCCP compared to bafilomycin A_1_).
**Table S1:** Donor information for the nine experiments (EX #) including whether donor was in the young group (Y1–Y8) or old group (O1–O5), which protocol (BLSA, Baltimore longitudinal study of aging; ND, cytapheresis of volunteer donors; TP, tissue procurement for biomedical research), age in years, and sex (female or male).

## Data Availability

The data used to support the findings of this study is available from the corresponding author upon request.
