# Supplementary material for: Preservation of Autophagy May Be a Mechanism Behind Healthy Aging
Source: Aging Cell. 2025 Oct 13;24(11):e70246. doi: 10.1111/acel.70246 (PMC12611306; doi:10.1111/acel.70246)
Supplement: Supplementary file 1 — Figure S1: Additional analysis of basal autophagic activity in CD4+ T cells from young and old healthy donors. (a) Analysis of LC3‐positive compartments (autophagosomes and autolysosomes) for area per cell in untreated cells. (b) For each donor within individual experiments, LC3 puncta per cell values of basal (untreated). (c) Analysis of LC3‐positive compartments (autophagosomes and autolysosomes) for area per cell in bafilomycin A1‐treated cells. (d) For each donor within individual experiments, LC3 puncta per cell values of basal (untreated) compared to bafilomycin A1‐treated compartments. (e) Analysis of LC3 flux, expressed as the ratio of LC3 area per cell before and after bafilomycin A1 treatment. f, for each donor within individual experiments, LC3 flux (autophagy flux), expressed as the ratio of LC3 puncta per cell after bafilomycin A1 treatment to before (untreated). (g) Analysis of net LC3 flux, expressed as the difference between LC3 area per cell of bafilomycin A1‐treated cells and untreated cells. (a–g) The number of independent experiments is nine with each experiment containing one young and one old donor with the exception of experiment 7, which has two young donors and one old donor. p values were calculated using a likelihood ratio test to compare this model against a null model without the age variable using ANOVA. *p < 0.05. (h) Mixed effect sensitivity analyses of LC3 flux (autophagy flux), expressed as the ratio of LC3 puncta per cell (left) or LC3 area per cell (right) after bafilomycin A1 treatment to before (untreated) with experiment 1 removed (experiment 1 includes the 93 years old male outlier). The number of independent experiments is eight with each experiment containing one young and one old donor with the exception of experiment 7, which has two young donors and one old donor. p value was calculated using a likelihood ratio test to compare this model against a null model without the age variable using ANOVA. (i) Mixed effect sensitivi [file ACEL-24-e70246-s001.pdf]

Figure S1

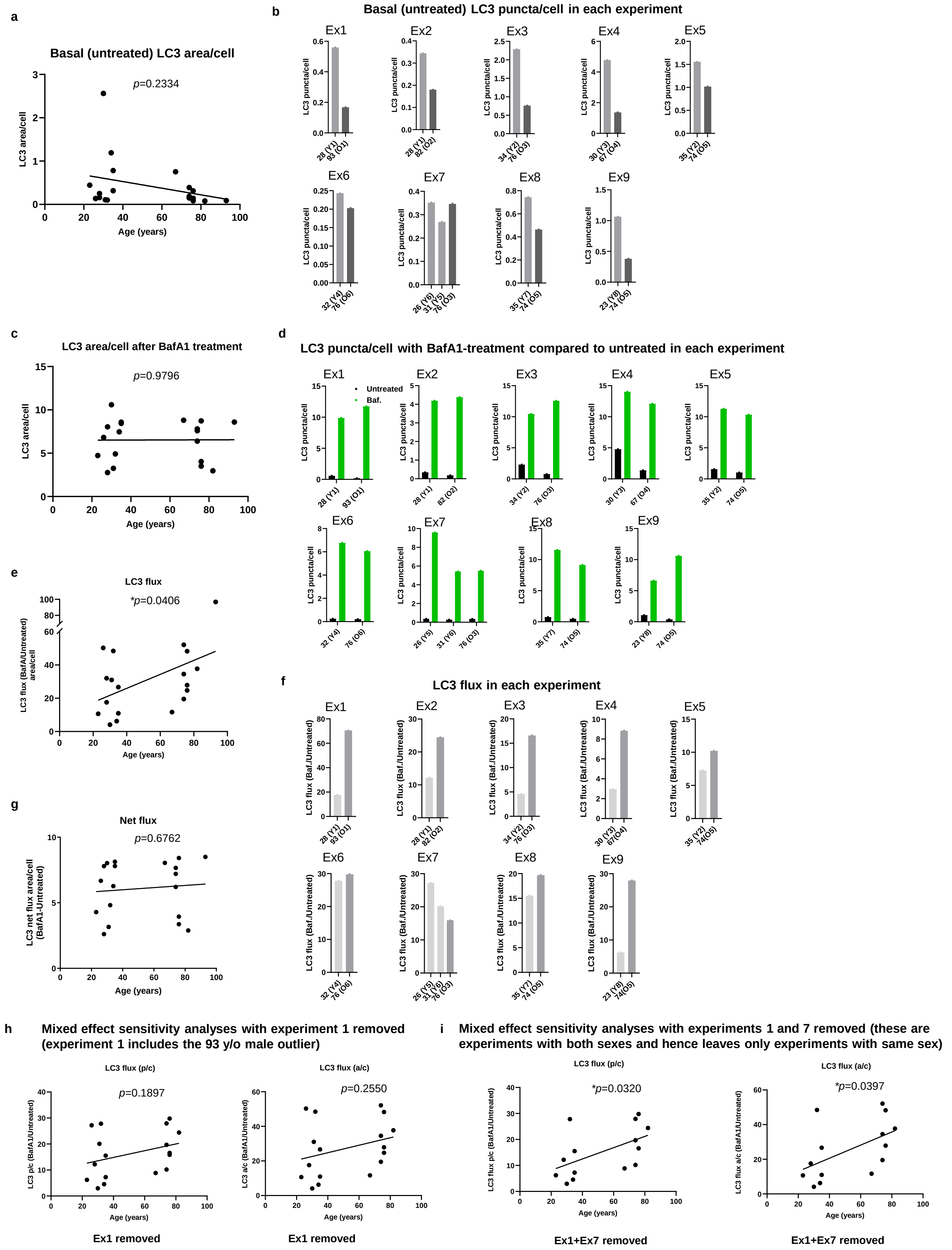

a
Basal (untreated) LC3 area/cell
p=0.2334
LC3 area/cell
Age (years)
b
Basal (untreated) LC3 puncta/cell in each experiment
Ex1
Ex2
Ex3
Ex4
Ex5
Ex6
Ex7
Ex8
Ex9
LC3 puncta/cell
28 (Y1)
93 (O1)
82 (O2)
34 (Y2)
76 (O3)
30 (Y3)
67 (O4)
35 (Y2)
74 (O5)
32 (Y4)
76 (O6)
26 (Y6)
31 (Y5)
35 (Y7)
23 (Y8)
c
LC3 area/cell after BafA1 treatment
p=0.9796
LC3 area/cell
Age (years)
d
LC3 puncta/cell with BafA1-treatment compared to untreated in each experiment
Untreated
Baf.
LC3 puncta/cell
26 (Y5)
31 (Y6)
e
LC3 flux
*p=0.0406
LC3 flux (BafA/Untreated) area/cell
Age (years)
f
LC3 flux in each experiment
LC3 flux (Baf./Untreated)
g
Net flux
p=0.6762
LC3 net flux area/cell (BafA1-Untreated)
Age (years)
h
Mixed effect sensitivity analyses with experiment 1 removed (experiment 1 includes the 93 y/o male outlier)
LC3 flux (p/c)
p=0.1897
LC3 p/c (BafA1/Untreated)
Age (years)
Ex1 removed
LC3 flux (a/c)
p=0.2550
LC3 a/c (BafA1/Untreated)
Ex1 removed
i
Mixed effect sensitivity analyses with experiments 1 and 7 removed (these are experiments with both sexes and hence leaves only experiments with same sex)
LC3 flux (p/c)
*p=0.0320
LC3 flux p/c (BafA1/Untreated)
Ex1+Ex7 removed
LC3 flux (a/c)
*p=0.0397
LC3 flux a/c (BafA1/Untreated)
Ex1+Ex7 removed

Figure S2

LC3 puncta/cell with CCCP-treatment compared to untreated in each experiment

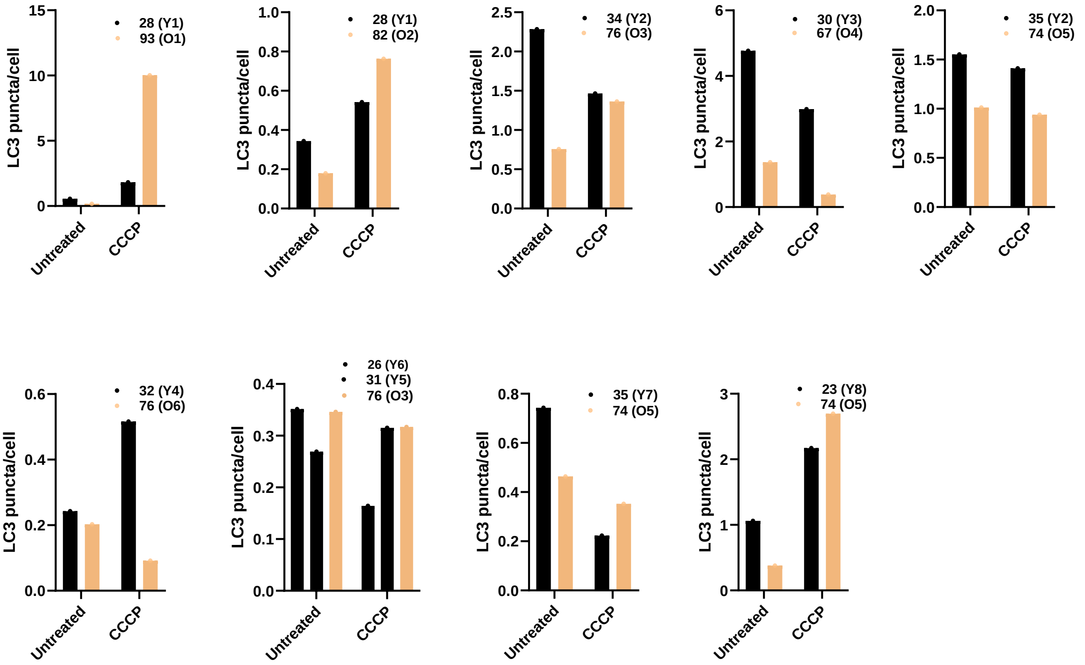

Figure S3

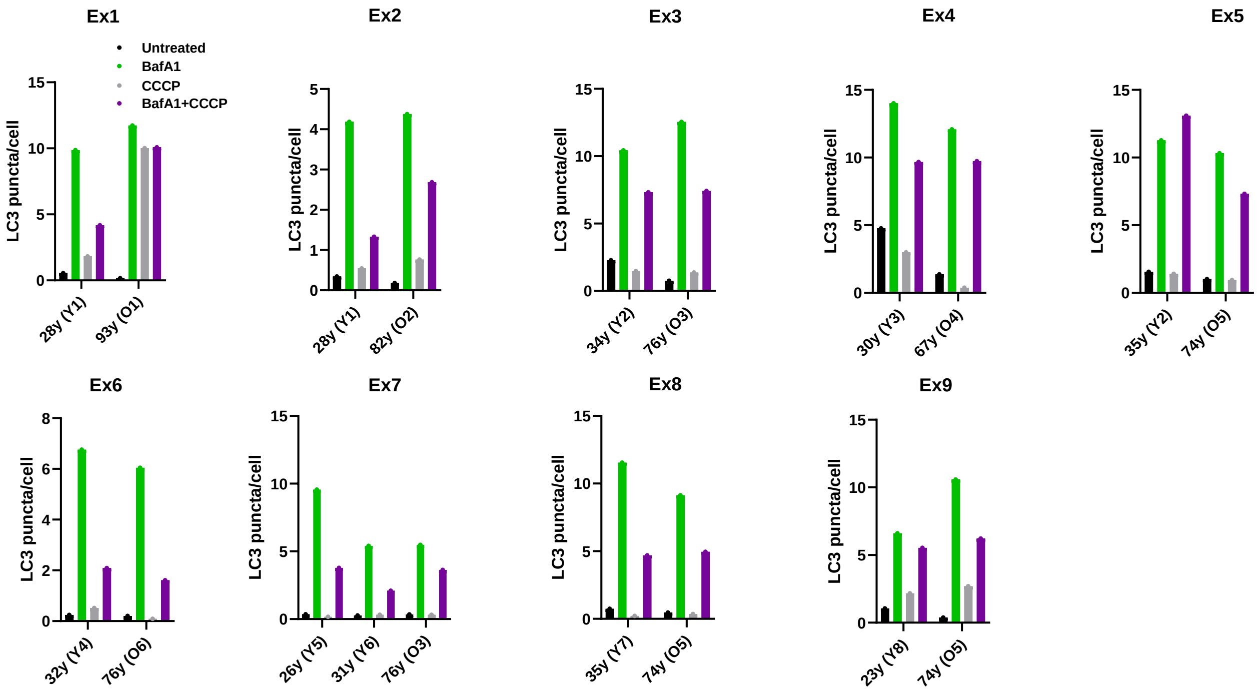

Ex1
Untreated
BafA1
CCCP
BafA1+CCCP
LC3 puncta/cell
28y (Y1)
93y (O1)
Ex2
28y (Y1)
82y (O2)
Ex3
34y (Y2)
76y (O3)
Ex4
30y (Y3)
67y (O4)
Ex5
35y (Y2)
74y (O5)
Ex6
32y (Y4)
76y (O6)
Ex7
26y (Y5)
31y (Y6)
76y (O3)
Ex8
35y (Y7)
74y (O5)
Ex9
23y (Y8)
74y (O5)

Supplemental Table

Donor Information

EX#	YOUNG	Protocol	AGE (y)	SEX	OLD	Protocol	AGE (y)	SEX
EX1	Y1	TP	28	F	O1	BLSA	93	M
EX2	Y1	TP	28	F	O2	BLSA	82	F
EX3	Y2	ND	34	M	O3	BLSA	76	M
EX4	Y3	ND	30	F	O4	ND	67	F
EX5	Y2	ND	35	M	O5	ND	74	M
EX6	Y4	TP	32	M	O6	BLSA	76	M
EX7	Y5	ND	31	F	O3	BLSA	76	M
	Y6	TP	26	F				
EX8	Y7	TP	35	M	O5	ND	74	M
EX9	Y8	TP	23	M	O5	ND	74	M
Total number of subjects	8 young				6 old			
